# Caregiving Experiences, Supportive Care Needs and Coping Strategies Among Family Caregivers of Patients with Colorectal Cancer in Kazakhstan: A Qualitative Descriptive Study

**DOI:** 10.3390/nursrep16080288

**Published:** 2026-08-18

**Authors:** Gulbakit Koshmaganbetova, Azamat Zharylgapov, Arip Koishybaev, Nauryzbay Imanbayev, Aliya Zhylkybekova

**Affiliations:** 1 Department of Evidence-Based Medicine and Scientific Management, West Kazakhstan Marat Ospanov Medical University, Aktobe 030000, Kazakhstan; g.koshmaganbetova@zkmu.kz; 2 Department of Oncology, West Kazakhstan Marat Ospanov Medical University, Aktobe 030000, Kazakhstan; a.koishybaev@zkmu.kz; 3 The Thoraco-Abdominal Oncology Department, Kazakh Oncology Institute, Almaty 050000, Kazakhstan; dr.imanbayevn@gmail.com; 4 Department of Pathological Physiology, West Kazakhstan Marat Ospanov Medical University, Aktobe 030000, Kazakhstan

**Keywords:** colorectal cancer, informal caregivers, family caregivers, qualitative study, caregiving experience, caregiver burden, stoma care, coping, nursing, supportive care needs, Kazakhstan

## Abstract

**Background:** Family caregivers play a central role in supporting people with colorectal cancer (CRC) and often manage complex physical, emotional, and practical demands. However, evidence regarding their experiences and supportive care needs in Kazakhstan remains limited. This qualitative study explored caregiving experiences, caregiving burden, caregivers’ needs, and coping strategies. **Methods:** A qualitative descriptive design was used, involving semi-structured interviews with 21 family caregivers caring for patients with CRC. Participants were recruited purposively from the Medical Center of West Kazakhstan Marat Ospanov Medical University and outpatient clinics in Aktobe between December 2025 and March 2026. Interviews were audio-recorded, transcribed verbatim, and analyzed using inductive reflexive thematic analysis. **Results:** Most family caregivers of patients with colorectal cancer were women (95.2%). Five main themes developed: emotional challenges, transformation of daily life, caregiving tasks, caregivers’ needs and support gaps, and coping strategies and resilience. Diagnosis was described as a distressing experience characterized by shock, fear, and uncertainty. Caregiving substantially disrupted employment, financial stability, and family roles, often requiring work adjustment or leaving the workforce. Caregivers reported insufficient preparation for stoma care and expressed a strong need for structured training. Social isolation was common, as both caregivers and patients experienced a shrinking of their social support networks. Despite substantial burden, caregivers described adaptive responses to ongoing emotional and practical demands, and resilience was a prominent theme. **Conclusions:** Family caregivers of patients with colorectal cancer in Kazakhstan face interconnected emotional, informational, physical, and system-level challenges, while also drawing on resilience. The findings highlight priorities for support, including structured stoma care education, psychological services, recognition of caregivers’ roles, and improved discharge and transitional care.

## 1. Introduction

Colorectal cancer (CRC) is the third most commonly diagnosed cancer and the second leading cause of cancer-related mortality worldwide, with more than 2.0 million new cases and approximately 918,000 deaths estimated in 2024 [1]. Kazakhstan reflects this global pattern, with approximately 3500 new cases annually and incidence and mortality rates of 14.9 and 7.1 per 100,000 population, respectively [2].

A diagnosis of CRC affects not only patients but also those close to them, including family members and friends. In addition to providing social and emotional support, family members often have to take responsibility for multiple aspects of patient care [3]. The term “family caregivers” refers to informal, unpaid relatives who provide physical, emotional, practical, or healthcare-related support to a person with CRC [4]. McMullen et al. described them as “health managers”, because they perform complex health-management activities, coordinate care, and respond to patients’ changing needs [5]. In Kazakhstan, healthcare workforce shortages, increasing demand for cancer care, and pressure on oncology and palliative care services may limit the capacity of formal services to address all patient and caregiver needs across treatment, survivorship, palliative care, and home care [6,7,8]. In this context, family caregivers often take on responsibility for meeting patients’ physical, emotional, informational, social, and practical needs throughout the cancer care trajectory [7,8]. Oncology nurses therefore play an important role in educating patients and family caregivers, preparing them for discharge, teaching stoma care skills, and coordinating the transition from hospital to home [9,10,11].

Patients with CRC often undergo prolonged and multimodal treatment [12,13,14]. This treatment can place substantial demands on patients and their family caregivers. The combination of caregiving responsibilities, emotional strain, disruption of daily life, and financial pressures may contribute to caregiver burden [15,16].

Caregiver burden refers to the perceived adverse effects of caregiving on a caregiver’s emotional and physical health, social relationships, daily activities, and financial circumstances [17]. It encompasses both objective demands, such as the time, tasks, and financial costs associated with caregiving, and subjective responses, including distress, worry, exhaustion, and a perceived loss of control. Among caregivers of patients with CRC, caregiver burden may vary across the treatment trajectory and may be particularly pronounced during intensive treatment periods [16,18]. Persistently high burden can contribute to fatigue, disruption of daily life, social isolation, financial difficulties, reduced quality of life, and symptoms of anxiety and depression [18,19]. Caregiver burden may also affect patients indirectly by limiting caregivers’ capacity to provide complex care at home. Many informal cancer caregivers undertake clinical and care-management tasks without adequate training, while greater caregiver competence has been associated with lower unplanned healthcare use and costs among patients with advanced cancer [20,21]. Patient and caregiver psychological health, self-care, and quality of life are interdependent, supporting a dyadic perspective on colorectal cancer care [19,22].

Supportive care needs include caregivers’ needs for informational, emotional, psychological, social, practical, financial, and healthcare support that enable them to maintain their own well-being and provide effective care. When these needs remain unmet, caregiver burden and psychological distress may increase, while caregivers’ preparedness and capacity to support patients may be reduced [23,24].

Coping refers to the cognitive and behavioural efforts used to manage demands that are appraised as taxing or exceeding the caregiver’s available resources [25]. Problem-focused strategies include seeking information, learning caregiving skills, and organising care. Emotion-focused strategies include seeking emotional support, expressing emotions, and engaging in spiritual practices, while meaning-focused strategies include acceptance, positive reframing, and finding meaning in the caregiving role [25,26,27,28]. Coping is dynamic and context dependent: some strategies may help caregivers adapt to changing demands, whereas avoidance, withdrawal, or ineffective communication may intensify psychological distress. Social support and constructive dyadic coping may help caregivers manage caregiving demands and reduce their perceived burden [19,28].

The caregiving trajectory in CRC is particularly demanding. Surgical resection with the creation of a colostomy remains a life-saving intervention for many patients with advanced or obstructive CRC [13,29,30]. After discharge, caregivers manage stoma care, often with limited preparation. They also cope with altered body image and potential stigma associated with bowel dysfunction, coordinate complex nutritional requirements, and maintain emotional resilience throughout cycles of treatment, recurrence, and uncertainty [29,30,31].

Qualitative studies from diverse contexts have identified emotional shock at diagnosis, insufficient information regarding stoma care, increased caregiving burden, social isolation, and the search for meaning in caregiving experiences [32,33,34]. Much of the existing evidence has been generated in high-income settings [35]. The availability and quality of healthcare vary across countries depending on their economic resources and health-system capacity [7]. Nevertheless, healthcare systems across different socioeconomic contexts increasingly face common pressures, including population ageing, a rising cancer burden, workforce shortages, increasing care costs, and growing reliance on families for the provision of care [35,36]. Little is known, however, about how family caregivers of patients with colorectal cancer experience these challenges in Central Asian settings. In Kazakhstan, family structures, cultural expectations regarding caregiving, and the organisation and availability of healthcare resources may shape caregiving experiences [7,37,38]. At the same time, many caregiving challenges may be shared across different healthcare systems [35]. To our knowledge, no qualitative study has explored caregiving experiences, caregiver burden, supportive care needs, and coping strategies among family caregivers of patients with colorectal cancer in Kazakhstan. Therefore, this study aimed to explore the caregiving experiences, caregiver burden, supportive care needs, and coping strategies of family caregivers of patients with colorectal cancer in Kazakhstan.

## 2. Methods

### 2.1. Study Design

This study employed a qualitative descriptive design and used semi-structured interviews with family caregivers of patients with colorectal cancer. This design was selected to provide a clear, experience-near description of caregivers’ perspectives grounded in their accounts and to generate practice-oriented insights relevant to applied health research [39]. This study was reported in accordance with the Consolidated Criteria for Reporting Qualitative Research (COREQ) guidelines [40]. The completed COREQ checklist is provided as Supplementary Table S1.

### 2.2. Research Team and Reflexivity

The interviews were conducted by the first two authors, G.K. (PhD) and A. Zharylgapov (MD, PhD candidate), both affiliated with West Kazakhstan Marat Ospanov Medical University, Kazakhstan. At the time of the study, G.K. was a faculty member and researcher with extensive experience in qualitative health research and previous publications using qualitative methodologies. A. Zharylgapov was a doctoral researcher who had completed formal training and coursework in qualitative research methods, qualitative interviewing, and thematic analysis. G.K. is female and A. Zharylgapov is male. Both interviewers underwent additional preparation before data collection to ensure consistency in interviewing techniques and adherence to qualitative research standards. A. Zharylgapov had previous clinical experience working with patients diagnosed with colorectal cancer. His clinical experience facilitated an understanding of the caregiving context but could also have influenced data interpretation. This potential influence was acknowledged through reflexive notes, open-ended questions, team discussions of the codes, and comparison of the interpretations with the original transcripts. No prior relationship existed between the researchers and participants before recruitment. Before each interview, participants were informed about the researchers’ professional backgrounds, their roles in the study, and the purpose of the research.

### 2.3. Setting and Participants

The study was conducted between December 2025 and March 2026. Participants were recruited using purposive sampling in the departments of palliative care and oncological surgery at the Medical Center of West Kazakhstan Marat Ospanov Medical University and outpatient polyclinics (Aktobe city).

Recruitment was conducted through telephone calls and face-to-face invitations by members of the research team. A total of 21 family caregivers (FCGs) of individuals diagnosed with colorectal cancer participated in the study. Family caregivers were eligible if they were aged 18 years or older, identified themselves as the primary caregiver of a person diagnosed with colorectal cancer, and had been actively involved in caregiving, with no restriction on the duration of caregiving. Participants were required to be able to communicate in the language used for the interviews and provide informed consent. During recruitment, four family caregivers declined participation without providing a reason. No participants withdrew after enrolment.

### 2.4. Data Collection

The semi-structured interview guide was developed in accordance with the study objectives and informed by LeSeure and Chongkhamang’s systematic review and meta-synthesis of cancer caregiving experiences, as well as previous qualitative studies examining family caregivers’ experiences, caregiving challenges, social support, positive changes, and ostomy-related needs in colorectal cancer care [29,41,42,43,44,45]. Prior to the main study, the interview guide was tested with two family caregivers to assess the clarity, relevance, and comprehensibility of the questions. These pilot interviews were not included in the final analysis. Based on this pilot testing, minor wording adjustments were made to improve clarity.

The final interview guide is provided in Supplementary Table S2. It included the following broad questions: (a) Can you describe your experience of caring for a family member with colorectal cancer? (b) How did you feel when you first learned about your family member’s colorectal cancer diagnosis? (c) How, if at all, has your relationship with your family member changed since their diagnosis? (d) What challenges have you faced as a caregiver, and how have you managed or overcome them? What forms of support have been most helpful? (e) Can you describe your feelings when you first saw the stoma? What challenges did you experience in learning and providing stoma care? (f) In what ways has caregiving affected your personal life, family relationships, and work? Have there been any positive changes or personal benefits resulting from this experience? (g) What personal qualities or characteristics do you believe are important for someone caring for a person with colorectal cancer?

Interviews were conducted at a time and location convenient for participants, including private rooms at healthcare facilities or participants’ homes according to their preference. All interviews were held in quiet settings that ensured privacy and minimized interruptions, allowing participants to speak openly about their experiences. Before each interview, participants received written and verbal information regarding the study. Written informed consent was obtained from all participants. All interviews were audio-recorded with participants’ permission. Field notes were recorded during interviews. No individuals other than the participant and interviewer were present during the interviews. No repeat interviews were conducted. Transcripts were not returned to participants for comments and correction.

Interview duration ranged from 30 to 51 min. Data collection and analysis were conducted concurrently. After each set of interviews, the research team reviewed the depth, relevance, specificity, and variation of the dataset in relation to the study aim. Sample adequacy was assessed iteratively using the concept of information power, considering the focused study aim, the specificity of the participant group, the depth and quality of the interviews, and the diversity of caregiving experiences. The final sample of 21 participants was considered sufficiently information-rich to address the study aim and support the development of coherent themes [46,47]. All interviews were conducted in Russian or Kazakh according to participant preference and were transcribed verbatim in the language in which they were conducted. Once data analysis was complete, selected quotations were translated into English by one trilingual member of the research team and checked against the original by a second trilingual member to ensure that the intended and cultural meanings were retained.

### 2.5. Data Analysis

Audio-recorded interviews were transcribed verbatim and imported into MAXQDA Analytic Pro 26.2.1 (VERBI Software GmbH, Berlin, Germany). Data were analyzed using inductive reflexive thematic analysis, guided by Braun and Clarke’s six-phase approach (2006) [48,49]. The analysis was iterative and reflexive rather than a linear application of fixed analytic steps. Two researchers (A. Zhylkybekova and A. Zharylgapov) familiarized themselves with the transcripts and generated initial codes. Their involvement was intended to broaden interpretative perspectives rather than establish intercoder agreement. Initial codes and developing themes were subsequently discussed during regular analytical meetings. These discussions were used to challenge interpretations, deepen analytical insights, and refine the developing themes throughout the iterative analytic process. Transcripts were not returned to participants, and participants were not invited to review or validate the preliminary findings or final themes.

### 2.6. Trustworthiness

To support the trustworthiness of the findings, we followed Lincoln and Guba’s (1985) criteria for establishing credibility, transferability, dependability, and confirmability [50]. Credibility was strengthened through prolonged engagement with the data, reflexive analytical discussions within the research team, and the use of rich verbatim quotations. Transferability was facilitated by detailed descriptions of the study context, participant characteristics (see Table 1), and the research setting. Dependability was supported by maintaining a detailed audit trail in MAXQDA, including reflexive memos and records of analytical decisions. Confirmability was promoted through ongoing researcher reflexivity and close engagement with participants’ accounts [49,51].

## 3. Results

Most caregivers were female with a mean age of 47.9 (SD = 12.1) years, of Kazakh ethnicity, and married. The majority of care recipients were male with a mean age of 60.5 years (SD = 11.1). Spouses represented the largest caregiver group (*n* = 14). Detailed participant characteristics are presented in Table 1.

Five themes and 19 subthemes were generated: (1) emotional challenges, (2) transformation of daily life, (3) caregiving tasks, (4) caregivers’ needs and support gaps, and (5) coping strategies and resilience. Detailed thematic structure is presented in Table 2.

### 3.1. Emotional Challenges

All family caregivers described receiving the diagnosis as a distressing experience accompanied by shock, panic, fear, helplessness, and concern about losing their loved one. Many participants reported an initial period of disbelief, denial, and difficulty accepting the diagnosis. These emotional responses were particularly pronounced immediately after the diagnosis and were frequently associated with uncertainty about the patient’s future.

*“When we first heard about the diagnosis, we were all shocked. I was scared. I thought it was all over. We all know that when you hear ‘tumor’ or ‘cancer,’ you think that’s it, the end.”*

*(FCG03)*

*“There was a fear of not knowing what to do and a fear of losing a loved one”*

*(FCG05)*

*“Of course, it was incredibly stressful because we didn’t expect this. We were in shock. It was terrible.”*

*(FCG04)*

*“There was a strong fear, and there still is. We never thought that something like this would happen to our family. I was at a loss, not knowing who to turn to.”*

*(FCG14)*

However, some caregivers declined professional psychological support, relying instead on inner strength, faith, or family:

*“I did not go to a psychologist. Prayer and family that is my support.”*

*(FCG16)*

Many family caregivers described crying as an immediate and uncontrollable response to overwhelming shock and grief. In some cases, emotional expression extended over several days and was experienced both individually and collectively within the family. For some participants, crying was described as a way of releasing emotional tension and coping with distress.

*“I sent my children to stay in the village with grandparents and cried for three days straight. We women express our emotions through tears.”*

*(FCG16)*

*“After we learned the diagnosis, we all sat in the car and cried together, my husband, our son, and I. When I feel overwhelmed, I go for a walk in the park, listen to music, cry, and calm down.”*

*(FCG14)*

*“All I did was cry.”*

*(FCG08)*

Caregivers’ emotional states gradually shifted from initial despair to the emergence of hope for recovery. They associated this change with reassurance from healthcare professionals regarding the disease stage and treatment options, as well as with observed improvements after treatment had begun.

*“Hope emerged after they learned that the cancer was at Stage II.”*

*(FCG16)*

*“At first there was only despair. Now I believe everything will be fine. The main thing is not to give up.”*

*(FCG15)*

*“Hope was restored when the endoscopist explained during the examination that the disease was treatable.”*

*(FCG14)*

*“Things probably became easier, and hope grew stronger after the first course of chemotherapy, when his condition began to improve.”*

*(FCG10)*

For some caregivers, this hope was preceded by a deep sense of acceptance rooted in religious faith, a finding discussed further in the theme of faith and spirituality.

### 3.2. Transformation of Daily Life

Caregivers reported that the caregiving process was accompanied by a substantial transformation of daily life, a reassessment of life priorities, and a shift in focus from their own needs to the needs of the patient. The illness also affected other family members, including children, which was reflected in changes in their behavior, increased responsibility, and greater emotional involvement.

*“The main thing is that he recovers. I no longer think about myself, all my strength goes to him.”*

*(FCG01)*

*“All I want is for my husband to recover and for this nightmare to be over. I came to realize that the whole atmosphere of the home depends on the woman. I cannot allow myself to be sad because my children and my husband look to me for strength.”*

*(FCG07)*

*“I also have a younger son who is 11 years old. He grew up almost overnight. He keeps asking, ‘How is Dad? Has Dad eaten?’ As a mother, I can see that he has become much more mature.”*

*(FCG15)*

Participants further noted a strong emotional interdependence between caregivers and patients, where emotional states were perceived as mutually influential. Improvement in the patient’s condition brought relief, whereas deterioration increased caregivers’ anxiety.

*“If he feels bad, I feel bad. If he smiles, I can breathe easily too.”*

*(FCG12)*

*“If I give up, my husband and children will give up too.”*

*(FCG07)*

*“You see that he is recovering, improving, and that things are already returning to normal.”*

*(FCG03)*

*“If I become depressed, my husband becomes depressed too, and if I break down, he does as well. If he breaks down, he cannot recover.”*

*(FCG10)*

Moreover, the demands associated with caregiving responsibilities disrupted caregivers’ employment trajectories and future life plans, while some participants had to modify their work schedules and adapt their roles in order to maintain work–family balance through flexible arrangements. These changes were frequently accompanied by considerable financial strain among caregivers. In addition, the illness contributed to a narrowing of social networks among both colorectal cancer patients and their caregivers, leading to social isolation. Some friends and relatives distanced themselves, uncertain about how to interact with individuals affected by the disease.

*“I quit my job completely. But I had already been planning to leave even before my husband got sick. I decided to find a job in a different field. However, because of my husband’s illness, I put those plans on hold.”*

*(FCG10)*

*“Our plans have changed dramatically, and we have become more mindful. We used to focus on material things and everyday plans, but all of that has faded into the background. Now, health comes first.”*

*(FCG07)*

*“We do not attend weddings of relatives and friends, but we do attend funerals, and only for a short time.”*

*(FCG18)*

*“The bags are expensive, the medications too. And I no longer work. How to survive—I do not know.”*

*(FCG04)*

*“Many friends simply stopped calling. They are afraid of the disease, afraid of saying the wrong thing.”*

*(FCG10)*

*“I was able to balance my family and work responsibilities. I moved my work online and supervised my child’s studies remotely.”*

*(FCG16)*

### 3.3. Caregiving Tasks

Day-to-day care meant washing, feeding, helping the patient move, and managing medicines. Participants described caregiving as a continuous and physically demanding process, particularly in cases where patients had limited mobility and required constant supervision and support. In addition to routine caregiving responsibilities, several caregivers reported independently performing dressings, injections, and stoma care procedures.

*“I clean the colostomy bag, change her clothes, and take her to the toilet. She wakes up at night and cannot sit up or walk independently. We first used a wheelchair, then a walker, and later a cane. We adjusted her diet, avoiding fried food and preparing only steamed meals. I remain in constant contact with the attending physician and receive guidance. I try to provide all the care as best as I can.”*

*(FCG16)*

*“In the hospital, I hardly ever left his side. I brought him water and assisted him in getting up, sitting down, and walking down the hallway.” (FCG07)*

*(FCG07)*

*“The care is constant. I clean the stoma, and when there is pain, I administer injections according to the physician’s prescription. For example, recently he had severe pain and developed a fever, and in such cases I give injections.”*

*(FCG17)*

Stoma care was identified as one of the most challenging tasks, especially in the first postoperative week. Caregivers described fear, skin complications, leakage, and odor. In addition to technical challenges, difficulties in stoma management were also associated with emotional stress and the need for gradual adaptation by both patients and caregivers.

*“I was afraid to even touch it. When the bag leaked, I cried. I did not know what to do.”*

*(FCG02)*

*“Stoma care is also stressful, as it takes time to learn how to do it and to become accustomed to it.”*

*(FCG18)*

In addition to their primary caregiving responsibilities, caregivers also provided emotional support and encouraged patients to continue treatment. In some cases, they encountered resistance to treatment, including refusal of surgery, chemotherapy, and other medical interventions, and made efforts to persuade patients to continue treatment, particularly during the psychological crisis following diagnosis.

*“He did not want the surgery. I had to persuade him for a long time, explaining that this was the only chance.”*

*(FCG06)*

Some caregivers used humor and references to family responsibilities to encourage patients to continue treatment.

*“I joked with him: it’s easy for you, you’ll be resting peacefully, and I’ll be left struggling to raise four children on my own. Aren’t you going to marry off the children yourself? This is life, you cannot simply give up; you are not the only one this is happening to. You must fight, telling yourself: I will recover, my family and my children need me. That is how I lifted his spirits.”*

*(FCG11)*

*“She didn’t want to follow the doctor’s instructions, so I tried to make sure she followed all of them. I used a “carrot and stick” method. I ensured she complied with all the prescriptions, and at the same time, I could also indulge her and comfort her with warm, affectionate words, calling her “mama.””*

*(FCG16)*

Many caregivers, particularly women, are left with responsibilities previously undertaken by the patient. Night-time caregiving and continuous physical demands resulted in marked exhaustion. Some participants described combining employment, intensive caregiving, and full household management, often with significant sleep disruption.

*“I go to work, I care for him, and I do everything around the house. I do not sleep at night, he calls, and I run.”*

*(FCG10)*

*“We are currently herding livestock, and I do all the herding myself. There is morning, midday, and evening herding, so you are with the livestock all day. My husband is unable to work due to shortness of breath and difficulty walking.”*

*(FCG17)*

*“In addition to caring for my father-in-law, I have six children at home, as well as elderly family members who also require care.”*

*(FCG06)*

### 3.4. Caregivers’ Needs and Support Gaps

Most caregivers reported that training in stoma care in hospitals was insufficient and delivered too quickly. Participants emphasized the need for more detailed and accessible educational formats, including brochures, video materials, individual consultations, and home-based training. In the absence of professional guidance, caregivers relied on online resources and the experiences of other patients.

*“We need brochures, videos, someone to come to our home and show us again. Once in the hospital is not enough.”*

*(FCG18)*

*“They showed me once or twice, quickly, and I was so stressed that I remembered nothing. At home I had to figure it out myself from YouTube videos.”*

*(FCG09)*

*“At hospital discharge, there should be a dedicated specialist who provides emotional preparation and explains everything in detail. Nurses often demonstrate stoma care quickly, assuming it is clear, but in a state of stress patients do not hear or remember the information.”*

*(FCG07)*

*“YouTube became my salvation. I found videos on how to change the bag. The hospital did not show me that.”*

*(FCG05)*

### 3.5. Coping Strategies and Resilience

Coping strategies and resilience were mainly grounded in family support which functioned as a key resource for managing the complex challenges associated with caregiving.

*“My children have become my support. They help both morally and physically. I would not manage without them.”*

*(FCG07)*

*“I had support from my parents, siblings, and friends. They told me that everything would be alright and that there were good doctors who could help. The support I received from my family and friends reassured me and helped me feel calmer.”*

*(FCG12)*

In addition to their family support, caregivers also relied on their inner strength and character, which became especially evident during difficult life situations. This inner resilience helped them manage hardships, challenges, fears, and uncertainty.

*“A caregiver needs to have inner strength and confidence. Because when I became ill, my husband became frightened. He was worried that if I got sick, he would be left without care. One needs the right mindset. As they say, despair belongs to the devil.”*

*(FCG14)*

*“My personal approach is like this: I do not complain to psychologists or to anyone else. I may raise my voice or get angry, but then I forget about it. I am not the type of woman who stays depressed or constantly feels sorrow. I have strong resilience and endurance.”*

*(FCG15)*

*“I understood that, as a man, I needed to be calm and support them. I had to be firm, even stronger, in order to lift the overall spirit and mood and maintain positivity within the family.”*

*(FCG20)*

Some caregivers reported that religious faith helped them manage fear, uncertainty, and emotional distress.

*“It was about acceptance. I turned to the Almighty, to God. He is the best psychologist for me. I truly accepted it. I accepted my mother and my mother-in-law’s illness and I understand that you just have to fight, not give up. For me, acceptance came completely.”*

*(FCG21)*

*“Faith gives me strength. When it seems there is no way out, I pray and find calm inside. That is the main thing that keeps me going.”*

*(FCG11)*

## 4. Discussion

The study provides one of the first qualitative accounts of the experiences of family caregivers of people with colorectal cancer in Kazakhstan, offering insights from a Central Asian context where evidence on informal caregiving remains scarce [7,8,38]. Five interconnected themes were generated, highlighting that caregiving extended far beyond the provision of practical assistance and encompassed profound emotional, social, informational, and financial challenges. Caregivers described the diagnosis as a life-changing event that disrupted family roles, employment, and daily routines, while simultaneously assuming increasingly complex clinical responsibilities, particularly related to stoma care. Despite these demands, participants demonstrated considerable resilience, drawing primarily on family support and personal inner strength.

A key finding was that family caregivers perceived the diagnosis of colorectal cancer as a profound psychological turning point marked by fear, uncertainty, helplessness, and concerns about losing a loved one. These findings are consistent with previous qualitative evidence from Europe, North America, and Asia, where diagnosis is repeatedly described as an emotionally disruptive event that changes how caregivers view the future and initiates a prolonged process of psychological adaptation [26,41,42,43,44]. Similar experiences of shock, uncertainty, and anticipatory grief have been reported among caregivers of patients with colorectal cancer and other malignancies, suggesting that emotional distress appears to be a common component of the caregiving experience regardless of healthcare setting [35,41,42,44]. However, our findings also show that emotional responses were closely linked to uncertainty surrounding treatment outcomes and gradually shifted toward hope once caregivers perceived treatment as effective and the patient’s condition stabilized. This transition supports previous observations that emotional adaptation among caregivers is a dynamic rather than static process, with hope developing alongside increasing confidence in treatment and caregiving abilities [25,27,45].

This emotional adaptation did not occur in isolation but was accompanied by broader changes in caregivers’ everyday lives. As caregiving responsibilities increased, participants were required to reconsider their family roles, employment, personal priorities, and social relationships. Similar disruptions have consistently been described in studies of colorectal cancer caregivers, where caregiving frequently extends beyond assisting with treatment to becoming the central organizing principle of daily life [3,20,42,43,44]. Many participants described postponing employment plans, reducing work commitments, or leaving the workforce entirely to accommodate caregiving responsibilities, reflecting findings from previous studies showing that informal caregiving is associated with financial hardship and occupational disruption [3,20,35,42,52]. Social isolation also emerged as an important consequence, as both caregivers and patients withdrew from social activities because of physical limitations, treatment demands, and perceived stigma [29,30,34,42]. Notably, almost all caregivers in our study were women, reflecting the persistence of traditional caregiving norms in Kazakhstan [53,54]. Within our sample, caregiving responsibilities were predominantly assumed by women, consistent with previous reports of gendered caregiving patterns in Kazakhstan. Participants’ accounts further indicated that caregiving responsibilities often coexisted with household and emotional responsibilities within the family, potentially intensifying the overall caregiving burden [35,37,41,52].

Caregiving responsibilities associated with colorectal cancer were particularly complex, especially following stoma formation. Stoma care emerged as one of the most technically and emotionally demanding aspects of caregiving, requiring family members to manage appliance changes, hygiene, leakage, odor, and skin complications while also helping patients adjust to altered bodily functioning. This finding is consistent with previous qualitative studies showing that uncertainty regarding stoma management and fear of complications are major sources of distress for both patients and caregivers [5,29,30,32,33,34,55]. In addition to stoma care, participants frequently assumed responsibilities extending beyond conventional family support, including wound care, injections, medication management, and monitoring postoperative symptoms. These findings illustrate how complex clinical tasks may be transferred from healthcare professionals to family members, particularly when structured home-based nursing support is limited. Importantly, caregivers’ responsibilities were not confined to practical and clinical care [5,12,35,42]. They also played a central role in motivating patients to initiate and continue treatment, including persuading them to accept surgery or chemotherapy, encouraging them during periods of despair, and reminding them of their importance to the family [42,43,44]. Thus, family caregivers acted simultaneously as providers of technical care and as key sources of emotional encouragement and treatment engagement. This dual responsibility may place considerable pressure on caregivers, who are expected to manage demanding procedures while maintaining hope and psychological stability for the patient. The findings therefore reinforce the need to recognize family caregivers as integral members of the oncology care team and to provide them with structured stoma education, clear guidance on home-based clinical tasks, and communication and psychosocial support, so that responsibility for complex care and sustaining treatment motivation does not rest on families alone [9,10,11,23,24].

Despite experiencing considerable emotional and practical burden, caregivers demonstrated substantial resilience throughout their caregiving experience. Their coping resources were grounded in several interconnected sources, including support from family and friends, personal determination, patience, endurance, and religious faith. Practical and emotional assistance from children, parents, siblings, and friends helped caregivers feel less isolated and better able to sustain daily caregiving responsibilities. At the same time, participants emphasized the importance of inner strength, self-confidence, emotional control, and the ability to remain calm and solution-focused during periods of uncertainty. Previous qualitative studies have similarly identified resilience as an important protective process among caregivers of patients with colorectal cancer [26,35,41,43,45]. However, the present findings suggest that resilience should not be understood solely as an individual characteristic. Rather, it emerged through the interaction of personal resources, supportive relationships, and culturally meaningful coping practices [25,27,28,37,52]. For some participants, religious faith and spiritual practices represented important sources of comfort, acceptance, and emotional strength. Some caregivers perceived faith as sufficient emotional support or preferred it to professional psychological assistance. However, participants’ religiosity was not systematically assessed; therefore, these findings should not be interpreted as representative of all caregivers or of the broader religious culture in Kazakhstan. Importantly, some caregivers described concealing their own distress in order to remain strong for the patient and maintain emotional stability within the family. Although such emotional self-regulation may support caregiving in the short term, it may also mask unmet psychological needs and increase vulnerability to exhaustion over time [20,41,42,52].

The findings support a family-centered nursing approach that recognises caregivers as both individuals with supportive care needs and partners in care [4,9,11,23,24]. Key priorities include assessing caregiver preparedness, providing repeated stoma-care education and structured discharge preparation, monitoring caregiver strain during post-discharge follow-up, and facilitating referral to psychological or spiritual support according to individual and family preferences. Educational materials should also be available in Kazakh and Russian and provided in accessible formats [9,10,11].

Although these findings reflect the specific healthcare and sociocultural context of Kazakhstan, many of the underlying pressures are shared across healthcare systems. Population ageing, the increasing cancer burden, workforce shortages, rising healthcare costs, and growing reliance on family caregivers are also evident in high-income settings [1,6,35,36]. The need for long-term planning and sustained investment in caregiver preparation and support may therefore be relevant beyond the Central Asian context [11,24,36].

## 5. Limitations and Strengths

Several limitations should be acknowledged. First, participants were recruited from multiple healthcare settings within a single city in western Kazakhstan, which may limit transferability to caregivers in other regions. Second, the predominantly female sample underrepresents male perspectives, and the caregiving experiences and coping strategies identified may not apply equally to male caregivers. Third, caregiving duration varied widely, and some early post-diagnostic and post-operative experiences were recalled retrospectively and may have been influenced by subsequent adaptation. As each participant was interviewed once, changes in caregiving experiences over the illness trajectory could not be captured. Fourth, detailed stoma characteristics, including type, permanence, time since formation, and complications, were not systematically collected, limiting analysis of stoma-specific caregiving demands. Fifth, participants’ religious affiliation and religiosity were not assessed, so findings related to faith and spirituality should be interpreted as individual experiences rather than broader religious or cultural patterns. Sixth, although interviews were analysed in the original Kazakh or Russian and selected quotations were translated and cross-checked by trilingual researchers, some linguistic and culturally embedded meanings may have been attenuated in English. Finally, transcripts and final themes were not returned for member checking, so participants could not comment on or challenge the researchers’ interpretations. Despite these limitations, the study has several important strengths. To our knowledge, it is the first qualitative study in Kazakhstan to explore family caregiving in colorectal cancer, contributing evidence from an underrepresented Central Asian context. Purposive sampling across several clinical services enabled the inclusion of caregivers with varied caregiving durations and circumstances, while semi-structured interviews generated rich and detailed accounts. The cultural and linguistic competence of the research team was a further strength. All interviews were conducted and analysed directly in Kazakh or Russian by researchers fluent in both languages and familiar with the local context, allowing the analysis to remain closely grounded in participants’ original-language accounts.

### Implications and Future Research

The findings should be interpreted in light of the study limitations, particularly the urban setting and the predominance of female participants, reflecting prevailing caregiving patterns in Kazakhstan. Nevertheless, the study provides important insights into caregiving experiences in colorectal cancer within a Central Asian context, where such qualitative evidence has been largely absent. Future research should include quantitative studies using validated caregiver burden measures to examine the prevalence and correlates of caregiver burden across Kazakhstan regions. Longitudinal investigations are needed to document resilience trajectories over time and to examine how caregiving burden and coping strategies evolve across the cancer care continuum. Comparative research across Central Asian countries would help distinguish universal caregiving challenges from culture-specific factors. Equally important are intervention studies evaluating the effectiveness of structured stoma care education programs (e.g., home-based training, video materials, peer support groups) and accessible psychosocial support services tailored to resource-constrained settings.

## 6. Conclusions

In Kazakhstan, family caregiving for people with colorectal cancer extends beyond conventional family support and is an integral part of the cancer care process. Caregivers assume demanding emotional, practical, and clinical responsibilities, particularly following stoma formation, while frequently receiving limited preparation and formal support.

Although caregivers demonstrated considerable resilience, they drew heavily on informal family ties, personal resources, and faith while identifying important gaps in formal support. These findings underscore the need for greater integration of caregivers into colorectal cancer care through family-centred supportive care. Routine assessment of caregiver needs, structured stoma-care education, discharge preparation, and continued nursing support after hospital discharge should be embedded in clinical practice in order to improve both caregiver wellbeing and continuity of patient care.

## Figures and Tables

**Table 1:** Participants’ characteristics.

Characteristic	Care Recipient (*n* = 21)	FCG (*n* = 21)
Gender, *n* (%)		
Female	4 (19.0%)	20 (95.2%)
Male	17 (81.0%)	1 (4.8%)
Age, mean (SD)	60.5 (11.1)	47.9 (12.1)
Ethnicity, *n* (%)		
Kazakh	18 (85.7%)	18 (85.7%)
Russian	3 (14.3%)	3 (14.3%)
Marital status, *n* (%)		
Single	1 (4.8%)	2 (9.5%)
Married	19 (90.5%)	18 (85.7%)
Divorced		1 (4.8%)
Widowed	1 (4.8%)	
Education level, *n* (%)		
Secondary school	1 (4.8%)	3 (14.3%)
College	16 (76.2%)	10 (47.6%)
University	4 (19.0%)	8 (38.1%)
Caregiver relation to patient, *n* (%)		
Spouse/Partner		14 (66.7%)
Child (son and daughter)		4 (19.0%)
Other relative		3 (14.3%)
ECOG performance status, *n* (%)		
1	2 (9.5%)	
2	6 (28.6%)	
3	13 (61.9%)	
Disease stage *n* (%)		
Stage II	16 (76.2%)	
Stage III	4 (19.0%)	
Stage IV	1 (4.8%)	
Stoma, *n* (%)		
No	4 (19.0%)	
Yes	17 (81.0%)	
Stoma status *n* (%)		
Permanent	12 (70.6%)	
Temporary	5 (29.4%)	
Treatment context at the time of the interview, *n* (%)		
Post-treatment follow-up	11 (52.4%)	
Receiving or awaiting adjuvant chemotherapy	9 (42.9%)	
Receiving supportive care only	1 (4.8%)	

**Table 2:** Themes and subthemes derived from the analysis.

Theme	Subthemes	Representative Quotation
1. Emotional challenges	1.1 Emotional experiences of caregivers 1.2 Emotional expression through tears 1.3 Hope after accepting the diagnosis 1.4 Need for psychological support	*“When we first heard about the diagnosis, we were all shocked. I was scared. I thought it was all over…” (FCG03)*
2. Transformation of daily life	2.1 Self-sacrifice and reprioritization 2.2 Interdependence of patient and caregiver states 2.3 Changing of life plans and employment loss 2.4 Social isolation 2.5 Adaptation and adjustment over time	*“The main thing is that he recovers. I no longer think about myself, all my strength goes to him.” (FCG01)*
3. Caregiving tasks	3.1 Daily care obligations 3.2 The task of motivating the patient to undergo treatment 3.3 Stoma-related difficulties 3.4 Additional domestic duties	*“Stoma care is also stressful, as it takes time to learn how to do it and to become accustomed to it.” (FCG18)*
4. Caregivers’ needs and support gaps	4.1 Perceived information needs of caregivers 4.2 Challenges within the healthcare system 4.3 Financial burden and material needs of caregivers	*“At hospital discharge, there should be a dedicated specialist who provides emotional preparation and explains everything in detail. Nurses often demonstrate stoma care quickly, assuming it is clear, but in a state of stress patients do not hear or remember the information.” (FCG07)*
5. Coping strategies and resilience	5.1 Support from family and friends 5.2 Faith in God and spirituality 5.3 Resilience and inner resources	*“Faith gives me strength. When it seems, there is no way out, I pray and find calm inside. That is the main thing that keeps me going.” (FCG11)*

## Data Availability

The data that support the findings of this study are available on request from the corresponding author.

## Supplementary Materials

The following supporting information can be downloaded at: https://www.mdpi.com/article/10.3390/nursrep16080288/s1. Table S1: Consolidated Criteria for Reporting Qualitative Research (COREQ): 32-item checklist; Table S2: Questions for semi-structured interviews with family caregivers of patients with colorectal cancer.

## Public Involvement Statement

No public involvement in any aspect of this research.

## Guidelines and Standards Statement

This manuscript was reported in accordance with the COREQ for qualitative research.

## Abbreviations

CRC	colorectal cancer
FCG	family caregiver
