# Supplementary material for: Caregiving Experiences, Supportive Care Needs and Coping Strategies Among Family Caregivers of Patients with Colorectal Cancer in Kazakhstan: A Qualitative Descriptive Study
Source: Nurs Rep. 2026 Aug 18;16(8):288. doi: 10.3390/nursrep16080288 (PMC13516333; doi:10.3390/nursrep16080288)
Supplement: Supplementary file 1 [file nursrep-16-00288-s001.zip › nursrep-4422530-supplementary.pdf]

Table S1. Consolidated criteria for reporting qualitative studies (COREQ): 32-item checklist

Developed from:
Tong A, Sainsbury P, Craig J. Consolidated criteria for reporting qualitative research (COREQ): a 32-item checklist for interviews and focus groups. International Journal for Quality in Health Care. 2007. Volume 19, Number 6: pp. 349 – 357

**Item No**	**Guide Questions/Description**	**Reported on Page #**
**Domain 1: Research team and reflexivity**		
**Personal Characteristics**		
1. Interviewer/ facilitator	Which author/s conducted the interview or focus group?	Pg 4
2. Credentials	What were the researcher's credentials? E.g., PhD, MD	Pg 4
3. Occupation	What was their occupation at the time of the study?	Pg 4
4. Gender	Was the researcher male or female?	Pg 4
5. Experience and training	What experience or training did the researcher have?	Pg 4
**Relationship with participants**		
6. Relationship established	Was a relationship established prior to study commencement?	Pg 4
7. Participant knowledge of the interviewer	What did the participants know about the researcher? e.g. personal goals, reasons for doing the research?	Pg 4
8. Interviewer characteristics	What characteristics were reported about the interviewer/facilitator? e.g. Bias, assumptions, reasons and interests in the research topic	Pg 4
**Domain 2: study design**		
**Theoretical framework**		
9. Methodological orientation and Theory	What methodological orientation was stated to underpin the study? e.g. grounded theory, discourse analysis, ethnography, phenomenology, content analysis	Pp. 4–5
**Participant selection**		
10. Sampling	How were participants selected? e.g., purposive, convenience, consecutive, snowball	Pg 4
11. Method of approach	How were participants approached? e.g., face-to-face, telephone, mail, email	Pg 4
12. Sample size	How many participants were in the study?	Pg 4
13. Non-participation Setting	How many people refused to participate or dropped out? Reasons?	Pg 4
14. Setting of data collection	Where was the data collected? e.g., home, clinic, workplace	Pg 5
15. Presence of nonparticipants	Was anyone else present besides the participants and researchers?	Pg 5
16. Description of sample	What are the important characteristics of the sample? e.g. demographic data, date	Pg 6, Table 1
**Data collection**		

**Item No**	**Guide Questions/Description**	**Reported on Page #**
17. Interview guide	Were questions, prompts, and guides provided by the authors? Was it pilot tested?	Pp. 4–5
18. Repeat interviews	Were repeat interviews carried out? If yes, how many?	Pg.5
19. Audio/visual recording	Did the research use audio or visual recording to collect the data?	Pg.5
20. Field notes	Were field notes made during and/or after the interview or focus group?	Pg.5
21. Duration	What was the duration of the interviews or focus group?	Pg 5
22. Data saturation	Was data saturation discussed?	Pg 5
23. Transcripts returned	Were transcripts returned to participants for comment and/or correction?	Pg 5
**Domain 3: analysis and findings**		
**Data analysis**		
24. Number of data coders	How many data coders coded the data?	Pg 5
25. Description of the coding tree	Did the authors provide a description of the coding tree?	Pg 5, Table 2.
26. Derivation of themes	Were themes identified in advance or derived from the data?	Pg 5
27. Software	What software, if applicable, was used to manage the data?	Pg 5
28. Participant checking	Did participants provide feedback on the findings?	Pg 5
**Reporting**		
29. Quotations presented	Were participant quotations presented to illustrate the themes/findings? Was each quotation identified? e.g., participant number	Pg 7-11
30. Data and findings consistent	Was there consistency between the data presented and the findings?	Pg 7-11
31. Clarity of major themes	Were major themes clearly presented in the findings?	Pg 7-11
32. Clarity of minor themes	Is there a description of diverse cases or a discussion of minor themes?	Pg 7-11

Table S2. Questions for semi-structured interviews with family caregivers of patients with colorectal cancer

No.	Questions
1.	Age of the respondent
2.	Gender of the respondent
3.	Education of the respondent
4.	Please tell me about your experience of caring for a family member with colorectal cancer, from the time of diagnosis to the present.
5.	How did you feel when you first learned about your family member's diagnosis?
6.	How did you come to take on the caregiving role, and what are your main caregiving responsibilities?
7.	How have your caregiving responsibilities changed over time, particularly during diagnosis, treatment, surgery, and recovery at home?
8.	What have been the most emotionally and physically difficult aspects of providing care? How have you managed these difficulties?
9.	Which tasks have been the most difficult?
10.	What information, advice, or training have you received from doctors, nurses, or other healthcare professionals? How useful and sufficient was this support?
11.	How prepared and confident do you feel in carrying out your caregiving responsibilities? What additional knowledge or skills would you like to develop?
12.	What role, if any, have you played in supporting the patient's treatment decisions or encouraging them to begin or continue treatment?
13.	How has caregiving affected your daily life, employment or studies, financial situation, family relationships, and social activities?
14.	What sources of practical, emotional, financial, or spiritual support have been available to you? How helpful have they been?
15.	What types of support or resources have been unavailable but would have made caregiving easier for you?
16.	What personal qualities, beliefs, or coping strategies have helped you continue providing care during difficult periods?
17.	What changes in healthcare or social support services would improve assistance for family caregivers of people with colorectal cancer?
18.	Considering your entire caregiving experience, what would be the most important and practical form of support for you and your family?
19.	Is there anything else about your caregiving experience that you consider important but that we have not discussed?
